# Ultrasound diagnostics of the abdominal aorta: English version

**DOI:** 10.1007/s00772-014-1411-1

**Published:** 2015-01-31

**Authors:** W. Schäberle, L. Leyerer, W. Schierling, K. Pfister

**Affiliations:** 1 Klinik für Viszeral-, Gefäß-, Thorax- und Kinderchirurgie, Klinik am Eichert Göppingen, Eichertstr. 3, 73035 Göppingen, Germany; 2 Gefäß- und endovaskuläre Chirurgie, Universitätsklinikum Regensburg, Regensburg, Germany

**Keywords:** Sonography, Aortic aneurysm, Measurement methods, Orthogonal measurement, Comparative study CT, Sonographie, Aortenaneurysma, Messmethode, Orthogonale Messung, Vergleichsuntersuchung CT

## Abstract

**Background and objectives:**

The ideal method for screening investigations is one which is as free as possible from side effects, is easily learnt, and can therefore be broadly used to detect abdominal aortic aneurysms (AAA) with a high degree of certainty. Although ultrasonography fulfils these criteria, the measurement method is not standardized. Different measurement methods are used in ultrasonography as well as in computed tomography (CT) studies and the measurement method is actually described sufficiently in only 57 % of cases.

**Methods:**

This article provides a critical review of the current literature on measurement methods and the validity of ultrasonography for the determination of aortic diameter, particularly in AAA, and presents the measurement principles for making measurements as precisely as possible.

**Results and conclusion:**

The most precise determination of aortic diameter is carried out by electrocardiogram (ECG) gating according to the leading-edge method with orthogonal slicing. Within the framework of screening investigations, sufficient measurement precision can be achieved by adherence to orthogonal slicing. Using these standardized measurement methods, ultrasonography shows valid and reproducible results even compared with CT and is the method of choice in screening investigations for AAA.

Electronic Supplementary Material is available for this article at 10.1007/s00772-014-1411-1 and accessible for authorised users.

## Abstract (de)

**Hintergrund und Fragestellung:**

Ideal für Screeninguntersuchungen ist eine Methode, die möglichst nebenwirkungsfrei, leicht erlernbar und somit breit einsetzbar mit hoher Treffsicherheit ein abdominelles Aortenaneurysma (AAA) erkennt. Obwohl die Sonographie diese Kriterien erfüllt, ist die Messmethode nicht standardisiert. Sowohl in Ultraschall- als auch in CT-Studien werden unterschiedliche Messmethoden angewandt und nur in 57 % der Fälle wird überhaupt die Messmethode ausreichend beschrieben.

**Methode:**

Kritisches Review der aktuellen Literatur zu Messmethodik und Validität der Sonographie bei der Durchmesserbestimmung der Aorta insbesondere beim AAA und Darstellung von Messprinzipien zur möglichst exakten Messung.

**Ergebnisse und Schlussfolgerungen:**

Die exakteste Durchmesserbestimmung wird EKG-getriggert, nach der Leading-edge-Methode mit orthogonaler Schnittführung (zur Gefäßachse) durchgeführt. Im Rahmen von Screeninguntersuchungen ist eine ausreichende Messgenauigkeit bei Einhaltung der orthogonalen Schnittführung erreicht.

Die Sonographie zeigt bei dieser standardisierten Messmethode auch im Methodenvergleich zum CT valide, reproduzierbare Ergebnisse und ist für Screeninguntersuchungen zum AAA die Methode der Wahl.

Additional material online This article includes an additional video on the visualization of an AAA with cardiac cycle-dependent diameter variations. This supplemental can be found at dx.doi.org/10.1007/s00772-014-1411-1.

Detailed description of the accompanying video Visualization of an AAA with cardiac-cycle-dependent diameter variation in real-time B-mode (*left*), while diameter variation (systolic/diastolic) is documented in time-motion mode over time (in the anteroposterior plane) (*right*).

## Introduction

Ultrasonography of the aorta is primarily performed to detect or exclude an abdominal aortic aneurysm (AAA). Since more than 90% of AAA are found in the infrarenal abdominal aorta, the occurrence of aneurysm rupture can generally be reduced by ultrasound screening in otherwise symptom-free patients [9]. Other aortic diseases, ranging from stenosis to acute occlusion (Leriche syndrome), embolism caused by thrombosis in peripheral occlusion, dissection, and aortitis are generally assessed in the context of a targeted diagnostic work-up due to relevant symptoms [20]. Ultrasound diagnosis of the aorta is free of side effects, fast, and cost-effective, as a result of which it has been broadly accepted, particularly for AAA screening. Once the examiner has undergone appropriate training, the method has a steep learning curve. Thus, when a suitable examination protocol is used, ultrasonography permits valid examination results that are highly accurate not just in special centers, but on a broad level. Measurement errors and measured value discrepancies compared with gold-standard CT occur due to failure to use standardized measurement methods [outer-to-outer (OTO) or inner-to-inner (ITI) diameter, measurement during systole or diastole, axial or orthogonal measurement plane); however, such errors and discrepancies occur in both examination modes, as discussed below.

## Technical equipment requirements and patient preparation

Due to its anatomical position anterior to the spine, adequate visualization of the aorta depends on an appropriate penetration depth. To this end, low-frequency curved transducers with a frequency of 2–5 MHz are used. In the case of extremely obese patients, extending the frequency range to 1 MHz is helpful.

The examination takes place with the patient in the supine position, the abdominal wall relaxed, and arms positioned parallel to the body. Other patient preparation measures are usually not required. Hampering artefacts caused by intra-abdominal air can be avoided by displacing the intestinal loops from medial to lateral, or compressing air-filled segments, by applying dosed pressure with the transducer. In addition to compressing intra-abdominal air, dosed pressure with the transducer can also reduce the distance from the skin to the aorta, thus improving visualization by reducing the penetration depth. This form of compression is contraindicated for pain reasons only in pre-operated patients with extensive adhesions.

## Examination procedure and particular aspects of the ultrasound diagnosis of aortic aneurysms

In screening programs, the aorta is visualized in transverse cross-section from the diaphragmatic hiatus to the aortic bifurcation, and its maximum diameter (orthogonal) and configuration described. More detailed complementary examinations assess the common, external, and internal iliac arteries in longitudinal and lateral sections for extent and morphology of stenosing and dilatative vascular lesions. An aortic aneurysm is present when the aortic diameter exceeds 3 cm, or in the case of a sudden doubling in aortic cross section at the point of largest diameter compared with aortic diameter in a proximally adjacent section.

Determining the longitudinal extent of an aneurysm is not relevant to treatment and is more likely to cause confusion in the numbers game. However, it is important to localize the aneurysm, including information on whether it begins in a supra- or infrarenal section, as well as on its infrarenal distance from the renal arteries and its extension in a peripheral direction, i.e., involvement of the common iliac artery and possibly the internal iliac artery. In addition to establishing the indication of the surgical method (endovascular approach, standard or special stent graft), a description of aneurysm morphology (thrombus) and the external iliac artery (access route) is relevant.

## B-mode ultrasound versus color duplex ultrasound

B-mode ultrasound (real-time gray-scale sonography) is the method of choice for AAA screening. Its accuracy in diagnosing aortic dilatation is virtually 100 % [1, 8, 12, 16, 24, 26]. The aorta is measured orthogonally at its greatest diameter in the anteroposterior (AP) and transverse planes. In addition, its topographical relationship to the renal artery branches, as well as any iliac vein involvement, needs to be assessed. Color duplex ultrasound is only required to distinguish the perfused lumen from wall thrombus and in the differential diagnosis of rare findings, such as inflammatory aortic aneurysm and aortitis (giant cell arteritis). This method also makes is easier to establish the topographical relationship of an aneurysm to the renal arteries, as well as its relationship to the internal iliac artery branch in the case of significant longitudinal extension of the aneurysm.

## Particular aspects of measurement methods

### Measuring point and cardiac-cycle dependence

Precise sonographic determination of the maximum diameter of an aortic aneurysm is decisive not only in establishing the primary indication to monitor or operate a patient, but is also relevant in the comparison of methods, i.e., with computed tomography (CT), in interobserver variance in sonography, and in maximum diameter follow-up. Measurement methods are not standardized either in sonography or in CT. Measurement inaccuracies arising due to varying measurement methods are often ignored [1, 4, 14].

**Fig. 1:**
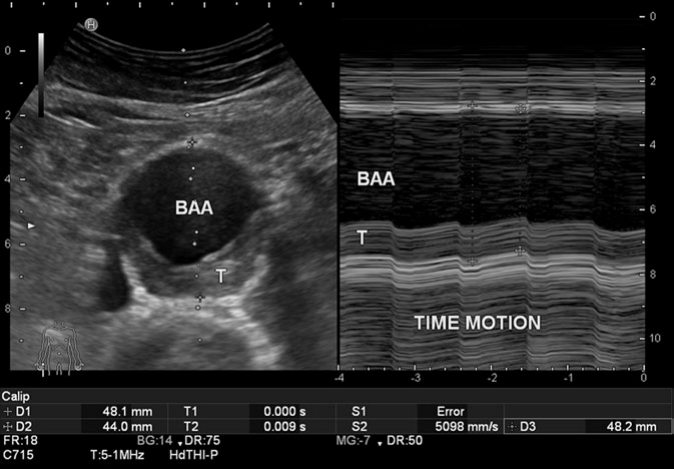
Diameter fluctuations from 48 mm (systolic) to 44 mm (diastolic) in time-motion modus (recording diameter change over time at the point of the sound beam visualized in B-mode). In B-mode cross section (*right*) with 48 mm diameter, incidental visualization in systole. Measurements made according to the leading-edge method (see video clip). *BAA* abdominal aortic aneurysm, *T* thrombus

When determining aortic diameter accurately, it must be borne in mind that fluctuations of 2– 4 mm in systolic/diastolic diameter are possible both with a normal aorta as well as in the presence of an aortic aneurysm (Fig. 1). There are only scant ultrasound studies that address the issue of cardiac cycle-dependent measurements [7], and in statistical CT measurements it is simply not possible to take these fluctuations into consideration for method-specific reasons [4]. This explains small variations both in monitoring as well as in method comparisons. Although ECG-gated measurement would be helpful [2, 3], this cannot be required of practice-based screening programs, nor is it practicable.

In addition, no specifications on diameter measurement are made in relation to aortic wall reflection [leading-edge (LELE) method] [4]. Thus, study results on OTO diameter determination [5, 8, 19] generally stand uncommented vis-à-vis those determining ITI diameter [11]. Although there appears to be good inter- and intraobserver variability with these measuring methods [4], ITI measurements inevitably underestimate diameter by 4 mm on average compared with OTO measurements [4].

**Fig. 2:**
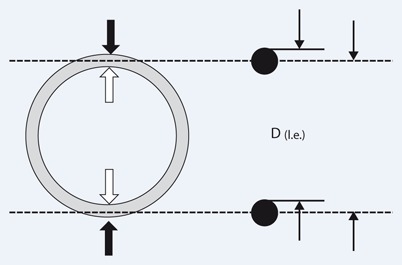
Measurement according to the outer-to-outer edge (*black arrow*), the inner-to-inner edge (*white arrow*), and the leading-edge method (*right*): outer wall reflection–inner wall reflection [*D(l.e.)*] of the opposing aortic wall in order to minimize and standardize the ultrasound overestimation of vessel thickness (*black dots*) caused by the blooming effect at boundaries with high acoustic impedance mismatches (such as vascular wall/blood). (Modified from [20])

It is known from serial measurements of other vessels and ultrasound phantoms that the LELE method (Fig. 1 and Fig. 2) yields the most accurate results, since the blooming effect causes an overemphasis of ultrasound reflections at boundaries with high acoustic impedance mismatches, as with vessel walls [20]. Measurements are then made from the outer wall reflection to the opposing inner wall reflection, i.e., from the outer start of the hyperechoic wall reflection to the point of reflection adjacent to the lumen on the opposing side (Fig. 2).

These particular aspects of the LELE method cause relevant yet not serious measurement inaccuracies (an overall maximum of 5–6 mm) and are therefore less relevant in primary aortic screening. They are however relevant when making the indication for surgery in borderline-sized AAA and monitoring (surgery is indicated if aneurysm diameter increases by 5 mm within 6 months). Also, when comparing ultrasound with CT, measurement errors can add up and result in statistically significant differences. In general, CT studies of AAA do not address the problems of precise AAA diameter determination described here.

### Transducer position and multiplanar reconstruction

**Fig. 3:**
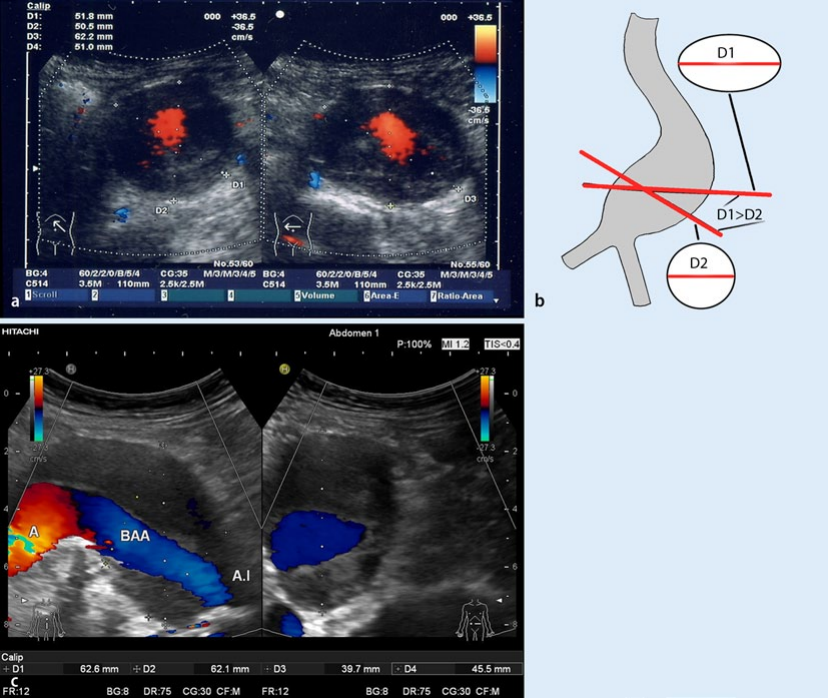
**a** Aortic diameter in AAA with left-lateral elongation: comparison of the measurement obtained in upper abdominal cross section (*right*; see body marker) of 62.2 mm (*D3*) with the measurement of 51.8 mm (*D1*) when the diameter is turned on the vessel axis at the same point (*left*). The orthogonal diameter measurement (*left*) corresponds to the real diameter. The anteroposterior (AP) measurement remains constant (50.5 and 51 mm in *D2* and *D4, respectively*). **b** Appropriate diameter measurement in AAA with an elongated vessel course. Measuring in abdominal cross section results in a false-high diameter due to the elliptical representation obtained in oblique section of the aneurysmal sack (*D1*). This measurement often also has low reproducibility, resulting in fluctuations in measured values. In order to obtain appropriate as well as reproducible measurements, the transducer should be turned in the area of maximum diameter in such a way that the real aneurysm transverse diameter (*D2*) perpendicular to the vessel axis is visualized (often a round structure) (modified from [20]). **c A comparison of **maximum AAA diameter **measur**ement (ventrolateral elongation) of 62.6 mm (*D1*) in abdominal cross section in the AP plane (*right*) with measurement in the orthogonal plane (perpendicular to the vessel axis in longitudinal section; *left*). The real aortic diameter here is only a maximum of 45.5 mm (*D4*); at the measurement point in the AP transverse plane, it is only 39.7 mm (*D3*). The measurement of 62.1 mm in cross section (*right*) is also marked with measuring marks (*D2*) in the longitudinal section (*left*). Diameter measurement in the AP plane in the *right-hand* section of the image corresponds to the AP measurement on CT in an axial plane (without reconstruction). *BAA* abdominal aortic aneurysm, *A.I* iliac artery

The measurement error produced by transverse cross-sectional upper abdominal determination in the case of an aortic axis visualized obliquely is more serious. Vessel dilatation often causes elongation. The aorta altered by an aneurysm tends to elongate with increasing size, generally following a curved course in a left-lateral direction, possibly also in a ventral direction. Where this is the case, maximum AAA diameter should not be determined ultrasonographically in transverse cross-section of the upper abdomen. This causes, as with axial CT measurement, an elliptical representation resulting in false-high measurements differing by up to 1–1.5 cm compared with the actual aneurysm measured using multiplanar reconstruction (Fig. 3 **a**, **b**, **c**). Therefore, the transducer must be turned in the area of greatest diameter in such a way that it is perpendicular to the aortic axis and/or that the ellipsis resumes a round structure in this area (Fig. 3 **a**, **b**). In the case of elongation of the aorta in a ventral direction, the diameter is best measured in the sagittal plane (Fig. 3 **c**).

Only by standardizing measurement method and transducer position in this way is it possible to monitor patients in an appropriate and reproducible manner. These factors need to be borne in mind in the case of differences in measurement values and when comparing methods (maximum cross-sectional diameter on CT).

3D ultrasound measurements or image fusion of ultrasound and CT are new and promising approaches to increasing measurement accuracy, particularly in monitoring studies [2, 3, 18].

## Method comparison: ultrasound vs. computed tomography

According to studies, a comparison of ultrasound and CT methods shows partially discrepant findings [21], often as a result of study design [1]. It should be noted that measurement method and transducer position are rarely specified in ultrasound studies. Of 23 studies [14], only 40 % provided information on the plane in which measurements were made. Only 30 % described the positioning of measurement marks, and only 10 % measured according to the LELE method. Despite a maximum point score of 4 for information on measurement method, an evaluation of studies yielded an average quality score of 2.5. An evaluation of the guidelines on aortic diameter measurement available in screening programs yielded an average score of only 1.6. On the other hand, CT AAA studies provide similarly imprecise information on measurement method, achieving an average quality score of only 1.6. Here again, measurement axis and position (outer or inner diameter) are often not defined [13, 14] and the correct measurement point rarely discussed [17]. Where measurement method is specified, it becomes apparent from a comparison of studies that distinct measurement principles have been used [14].

Upon comparison of CT and ultrasound, it is striking that the majority of studies [10, 14, 15, 22] suggest that ultrasound *underestimates* maximum aneurysm diameter compared with CT (often measured in the AP plane). A closer analysis of studies, however, reveals that the same often applies to transverse diameter on native axial CT without reconstruction. Two studies by Sprouse et al. [22, 23] document this measurement problem impressively. The first study shows that, in 95 % of cases, higher values were measured on axial CT compared with ultrasound measurements. At 5.69 ± 0.89 cm, the values on CT were significantly higher (*p* < 0.05) than on ultrasound at 4.74 ± 0.91 cm. In a follow-up study, however, Spouse then describes good concordance between ultrasound and CT measurements when the latter are based on reconstructions from orthogonal slicing. This method showed a mean difference of only 0.8 mm compared with ultrasound. In contrast, an internal CT comparison of AAA diameter determination showed significantly higher values in axial measurements at 58 mm on average (*p* < 0.05) compared with orthogonal measurements at 54.7 mm. The greater the aortic angulation, the higher the overestimation was in the axial measurement. This clearly demonstrates that these method-related measurement problems receive too little attention, not only in ultrasound examinations (Fig. 3 **b**) but also in CT measurements made in routine examinations as well as in the context of studies [14].

CT is generally considered the gold standard due to its lower examiner dependence and lower susceptibility to errors resulting from poor examination conditions. However, a consideration of the measurement problems discussed above renders the discussion on the gold standard in terms of technical equipment in aneurysm diagnosis secondary. It is more important at present to define a gold standard in terms of measurement method, a method according to which measurements and monitoring could be performed in a standardized manner and with which the most precise measurement of true aortic or aneurysmal diameter can be made [LELE method, standardized measurement in systole (or diastole), and orthogonal measurement].

## Conclusion

- B-mode ultrasound is the method of choice as a screening examination in the primary diagnosis of abdominal aortic aneurysm (AAA).
- It is *essential* for the method of aortic diameter measurement, as well as the measurement plane, to be standardized in order to enable comparative studies and valid monitoring examinations (ECG-gated, LELE method, and orthogonal slicing). In principle, however, this also applies to CT, which is considered the gold standard.
- Orthogonal reconstruction on CT, as well as 3D ultrasound and image fusion represent further approaches to improving comparability of CT and ultrasound.

## Electronic supplementary material

Video clip showing an AAA with cardiac cycle-dependent fluctuation of diameter (AVI 114MB)
